# Method for Detecting Core Malware Sites Related to Biomedical Information Systems

**DOI:** 10.1155/2015/756842

**Published:** 2015-03-03

**Authors:** Dohoon Kim, Donghee Choi, Jonghyun Jin

**Affiliations:** Agency for Defense Development, Daejeon 305-600, Republic of Korea

## Abstract

Most advanced persistent threat attacks target web users through malicious code within landing (exploit) or distribution sites. There is an urgent need to block the affected websites. Attacks on biomedical information systems are no exception to this issue. In this paper, we present a method for locating malicious websites that attempt to attack biomedical information systems. Our approach uses malicious code crawling to rearrange websites in the order of their risk index by analyzing the centrality between malware sites and proactively eliminates the root of these sites by finding the core-hub node, thereby reducing unnecessary security policies. In particular, we dynamically estimate the risk index of the affected websites by analyzing various centrality measures and converting them into a single quantified vector. On average, the proactive elimination of core malicious websites results in an average improvement in zero-day attack detection of more than 20%.

## 1. Introduction

Various types of cyber-attacks have recently been attempted on biomedical information systems [1, 2]. This is mainly because the personal records included in biomedical systems represent valuable financial information.

Unfortunately, current network security solutions are more vulnerable to advanced intelligent cyber-attacks [3] than to traditional cyber-attacks (e.g., distributed denial of service and spam). Because advanced persistent threat (APT) attacks [4, 5] are concentrated on the weak point of the target and the context, it is very hard to establish which APT attack detection method and defense system are most appropriate for biomedical information systems.

APT attacks are generally administered through malicious code exploit/landing/distribution sites, and infected User (or Administrator) PCs [6] easily give up contacts to biomedical information systems. Therefore, it is necessary to preisolate the contact points by which malicious code is disseminated, that is, the exploit/landing/distribution sites, to defend against these targeted attacks and protect biomedical information systems.

To defend against APT attacks on biomedical information systems, it is vital to analyze the way in which the network between medical websites and related websites is formed. This is because APT attacks make use of various sociotechnological methods [7] and create as many links as possible with medical service users (patients), medical staff, and related people via various contacts. Above all, administrators should detect malicious code targeted at biomedical information systems in an early stage and block the core-hub node in order to cope with APT attacks.

Therefore, this paper proposes a methodology that blocks and eliminates malicious code at an early stage by detecting the core-hub node at the root of the network between the biomedical information system-targeted malicious code exploit/landing/distribution site and the related websites. This paper also employs network analysis to estimate and manage the risk index of the detected malware sites by determining the potential risk factor of each exploit/landing/distribution point. In particular, we present a method for reprocessing malicious code so that it can be used as a reference in terms of malicious code detection and management.

Furthermore, this paper supports the efficient classification/application and management of massive blacklists in terms of biomedical information system-targeted malware sites. In this paper, we measure the risk index of websites with links to biomedical information systems and produce a malicious URL risk index (MRI) from this reference index.

## 2. Background

To detect the core-hub node, it is first necessary to understand the entire framework of malicious code distribution and infection through malicious websites. It is also important to understand the typical methods of detecting such websites and to appreciate certain risk estimation methods for the detection of malicious sites.

### 2.1. Malware Site Framework

**Figure 1:**
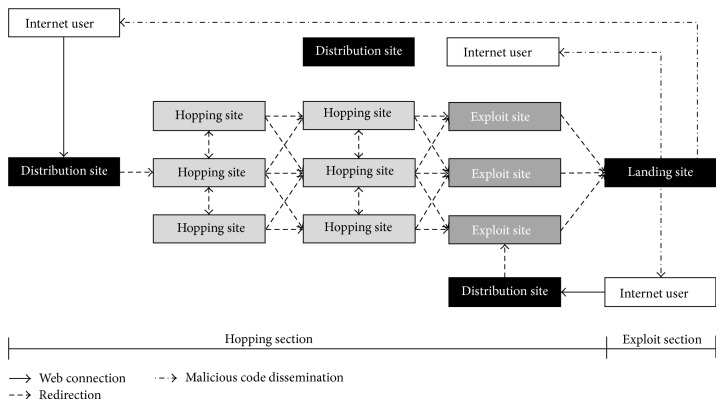
Definition of landing (or exploit)/distribution sites including malicious code.

To estimate the risk index of a malware site, we need to understand the dissemination route.  Figure 1 illustrates the definition and operation principles of the malware site detection framework, which is the basis for risk index estimation.

As shown in Figure 1, the victim (i.e., internet user) first visits the landing site connected with the distribution site and is then redirected to a hopping site or exploit site and finally downloads the malicious code. The internet user is eventually infected by the malicious code and may be damaged by various secondary cyber-attacks (e.g., personal information leaks, system destruction, and other host-derived attacks).

### 2.2. Web Crawling-Based Malicious Site Detection

Most studies on malware sites have mainly focused on detection. These studies primarily apply a web crawling method that rapidly collects the URL information of websites through a web crawler-based search engine [8, 9]. However, the web crawling technology used for malicious code collection selects and collects the execution files or compressed files that contain the malicious code, unlike the web crawling applied by search engines.

The web crawler considers URLs with file extensions of  .exe or HTTP headers with “application/octet-stream” content types to be execution files and downloads them. The crawler then inspects the headers of the downloaded files to confirm whether they are execution files. As execution files, compressed files and MS installation files are inspected and downloaded in the same way.

A number of web crawling-based automatic malicious code collection techniques have been proposed, most of which search websites via web crawling confirm whether the websites include malicious code and then download/analyze the relevant content.

## 3. Analysis of the Risk Index of Biomedical Information System-Related Malware Sites

We first propose a method for estimating the risk index of biomedical information system-targeted malware sites and estimate the ultimate risk index by analyzing the potential threat through a correlation analysis between the distribution site and the other connected sites.

The following sections describe our approach for predicting the risk index of the exploit/landing sites that redistribute the malicious code. The risk of individual exploit/landing sites is calculated through this prediction.

### 3.1. Vector-Based Risk Index Estimation Method

We employ a risk vector calculation to estimate the risk index [10]. As a planar vector is indicated by arranging two real numbers, a three-dimensional vector is indicated by arranging three real numbers in the rectangular coordinate system.

Spatial rectangular coordinates are indicated by arranging three real numbers that are orthogonal to each other through the origin *O*.

We fix the three coordinate axes *x*, *y*, and *z*, set the positive direction of the *x*, *y*, and *z* axes, and then define the length scale.

**Figure 2:**
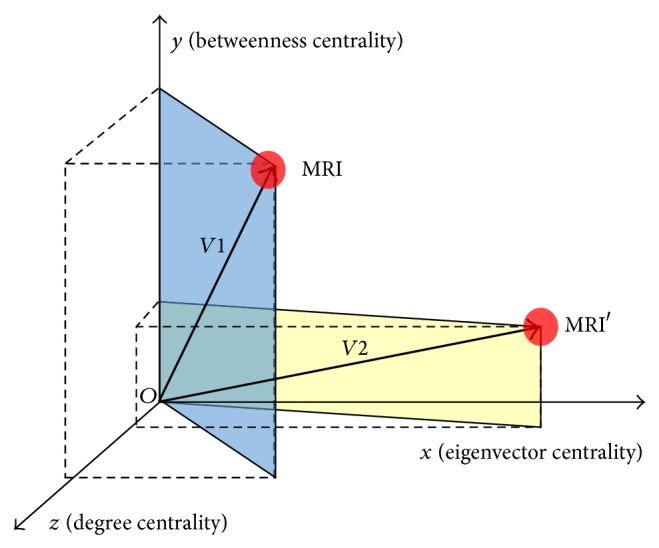
Entire analysis diagram for malicious code landing (or exploit)/distribution site risk estimation.

As shown in Figure 2, three vectors (connectivity, eigenvector, and betweenness) are used to estimate the risk index of malicious code landing (or exploit)/distribution sites, and the length is indicated by the vector sum [10]. The purpose is to indicate different vector values as lengths and then quantify the risk index through this.

We thus determine which sites have the highest-risk index and find the significance-based concentration degree of the corresponding sites by analyzing the central structure of the exploit/landing/distribution sites within malicious code that is connected to medical information systems. To interpret various meanings more objectively, this paper represents a risk factor and estimates the ultimate risk index by analyzing the connectivity [11–13], degree, eigenvector, and betweenness of the distribution site and exploit/landing site and vectorizing the calculated value. We now define each element of the risk index for the detected malicious code exploit/landing/distribution sites.

- *Degree Centrality Analysis of Nodes.* This is defined as the number of links incident upon a node. The degree can be interpreted in terms of the immediate risk of a node catching whatever is flowing through the network (such as malware sites). In the case of a directed network (where ties have direction), we usually define two separate measures of degree centrality, namely, the in-degree and out-degree centrality.
- *Eigenvector Centrality Analysis of Nodes.* This measures the influence of a node within a network. Relative scores are assigned to all nodes in the network based on the concept that connections to high-scoring nodes contribute more to the score of the node in question than equal connections to low-scoring nodes.
- *Betweenness Centrality Analysis of Nodes.* This is the number of shortest paths from all vertices to all others that pass through that node. A node with high betweenness centrality has a large influence on the transfer of items through the network, under the assumption that the transfer of items follows the shortest path.

### 3.2. Method to Estimate Malicious URL Risk Index (MRI)

**Figure 3:**
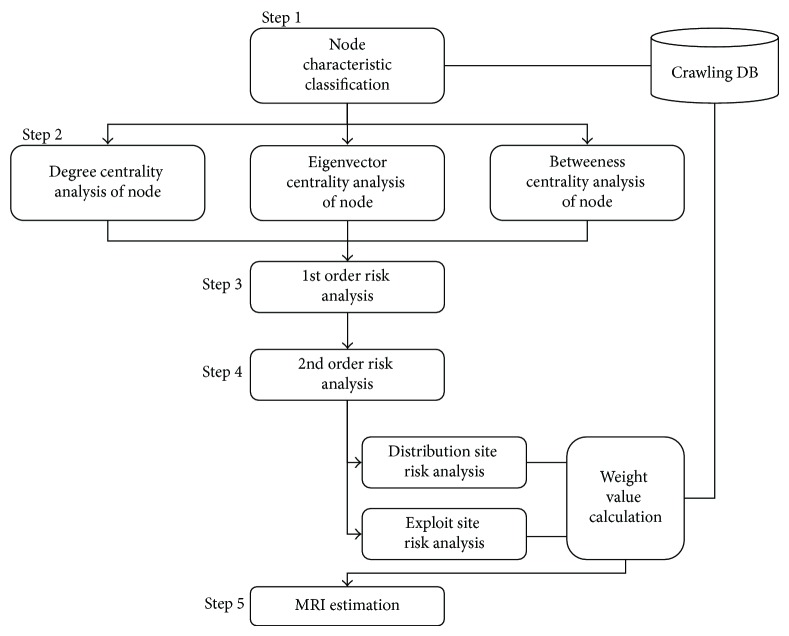
Entire analysis diagram for risk estimation of malicious code exploit/landing/distribution site.

To estimate the risk index of the URL of a malicious code exploit/landing/distribution site, we follow the process in Figure 3.

(1) *Step 1: Node Characteristic Classification.* Landing (or exploit)/distribution site information is classified by the logs produced through the self-developed malicious code detection crawler, and the detection history is sorted by time from the unit logs of the malicious code exploit/landing/distribution site. The basic risk is also estimated with the following log information.
  ① *Node Characteristic.* Whether the infected site is an exploit/landing site or a distribution site is confirmed. If there is no link to the detected malicious code (i.e., the information on the first infected site), the site is defined as a distribution site. If the URL of another site is exploited/distributed, the site is defined as an exploit/landing site.
  ② *Malicious Code Exploit/Landing/Distribution Site Information.* This is the URL of the detected malicious code exploit/landing/distribution site. The exploit/landing site can be the distribution site. If the distribution site is eliminated by a self-developed or other detection system, the exploit/landing site is rendered as the distribution site and operated continuously as a malicious code distribution site.
  ③ *IP Address, Country Code & Site Survivability.* Basic information is collected through the IP address and the related server location, and the current operating status is investigated. In particular, the survivability of the exploit/landing/distribution site is very important in estimating the risk index. Although the site has been treated or isolated and is no longer operated, the possibility of reinfection exists if the weak point is exposed continuously. Therefore, this should be reflected in the risk index estimation.
(2) *Step 2: Centrality Analysis of Node.* The following three indices are applied to the centrality analysis of each node.
  (i) Degree Centrality Analysis.
  (ii) Eigenvector Centrality Analysis.
  (iii) Betweenness Centrality Analysis.
  ① *Degree Centrality Index (DCI)*
    (i) A node that has more directly connected neighboring nodes has higher degree centrality. The scale of direct effects is measured.
    (ii) Degree centrality is calculated from the composition ratio of each node:
      (1) $$\begin{array}{c} \text{DCI}=\frac{\sum \left(\text{weight}\;\;\text{of}\;\;\text{incedent}\;\;\text{link}\right)}{\#\;\;\text{of}\;\;\text{nodes}-1},\\ \text{Time}\;\;\text{complexity:}\;\;O\left(n\right). \end{array}$$
  ② *Eigenvector Centrality Index (ECI)*
    (i) Assume that the number of the links included in node *N*_*j*_ is *l*_*j*_. If one of these links is connected to node *N*_*i*_, the probability that *N*_*j*_ passes *N*_*i*_ is 1/*l*_*j*_. Therefore, the ultimate ECI is as follows:
      (2) $$\begin{array}{c} \text{ECI}=I\left(N_{i}\right)=\frac{\sum I\left(N_{j}\right)}{l_{j}}. \end{array}$$
  ③ *Betweenness Centrality Index (BCI)*
    (i) To measure the BCI, measure the degree to which a node is located on the shortest route between nodes.
    (ii) The betweenness centrality of a node is higher if the node connects more different node groups. The BCI indicates the degree to which a node functions as a bridge in the entire network.
    (iii) It is possible to find the intermediate URL that links information between fields.
    (iv) Suppose that *g*_*jk*_ is the shortest possible route between nodes *j* and *k* in the network and *g*_*jk*_(*n*_*i*_) is the shortest possible route between nodes *j* and *k* that includes node *i*. The probability of the shortest route that includes node *i* is *g*_*jk*_(*n*_*i*_)/*g*_*jk*_
      (3) $$\begin{array}{c} \text{BCI}=C_{B}\left(n_{i}\right)=\frac{\sum_{j<k}g_{jk}\left(n_{i}\right)}{g_{jk}}. \end{array}$$
     If the main target node is constructed as a child node of depth 1, the degree will be increased. However, the BCI will be decreased by (3).
(3) *Step 3: 1st Order Risk Analysis.* The 1st order risk is estimated by calculating the Euclidean distance of the node analysis result from Step 2. The 1st order risk is thus estimated by the vector distance formula for the values calculated in Step 2:
  (4) $$\begin{array}{c} r_{1}=\sqrt{{\text{DCI}}^{2}+{\text{ECI}}^{2}+{\text{BCI}}^{2}}. \end{array}$$
(4) *Step 4: 2nd Order Risk Analysis*
  ① *Distribution Site Risk Analysis.* The risk index is estimated by considering the weights (overlapped infection history and survival ratio) based on the 1st order risk analysis. The distribution site risk is calculated by the vector of the values calculated in Step 3, the overlapped infection history (I) of each distribution site node, and the actual survival ratio (S).
  * Survival Ratio (*S*) is as follows: whether treatment has been given after infection (based on one year's information),
    (5) Treatment  Probability  S1 =Survival  CasesSurvival  Cases+Treatesd  Cases,Failure  Probability  S2 =Treated  CasesSurvival  Cases+Treatesd  Cases,r2=r1×I×S1 If  the  node  has  been  treated,r2=r1×I×S2 If  the  node  has  not  been  treated.
  ② *Exploit/Landing Site Risk Analysis.* The risk index is estimated by considering the weights (overlapped infection history and exposure frequency) from the 1st order risk analysis. The exploit site risk is calculated by the vector of values calculated in Step 3, the overlapped infection history (*I*) of each distribution site node, and the actual exposure frequency in a search website (*E*)
    (6) $$\begin{array}{c} r_{3}=r_{1}\times \left(\frac{2\times I\times E}{I+E}\right)\;\text{or}\;r_{3}=r_{1}\times I. \end{array}$$
(5) *Step 5: Malicious URL Risk Index (MRI).* The MRI is estimated from the 1st order risk analysis result and the risk index. The following formula can be deduced from the 1st order risk analysis result calculated in Step 3 and the risk index of each distribution/exploit site calculated in Step 4, considering the characteristics of the corresponding node:
  (7) $$\begin{array}{c} r_{\text{final}}=\sqrt{r_{1}^{2}+r_{2}^{2}+r_{3}^{2}}. \end{array}$$

## 4. Experimental Results

We conducted experiments to examine the performance of our zero-day detection method based on MCC.

For these experiments, we processed the detection log acquired by crawling biomedical information system-related malware sites with the developed MCC in the log form stated in Step 1.

The estimated risk values are intuitive in our proposed model. That is, our final interpretation is based on the crawling result. Additionally, the crawling method uses a blacklist or known patterns. Thus, our proposed model exhibits a low false positive rate.

The MCC detection method proceeds as follows. The attacker (hacker) inserts malicious code into a specific webpage by operating a malicious code distribution server on the internet or by hacking a vulnerable web server. The clients (or users) of the web server involuntarily use the exploit/landing/distribution site containing the malicious code and download the malicious code. Eventually, the attacker collects the client accounts and various other information from the infected server and proceeds to act maliciously.

The proposed system searches/crawls 2.5 million sites on a continuous basis, detects/blocks the inserted malicious code, and establishes/operates a malicious code blacklist.

### 4.1. Analysis Results

As a post hoc study based on the results of the MCC operation for a specific period, our results support decision making for proactive responses and follow-up measures, enabling biomedical information system security experts or administrators to maximize their operational efficiency.

**Figure 4:**
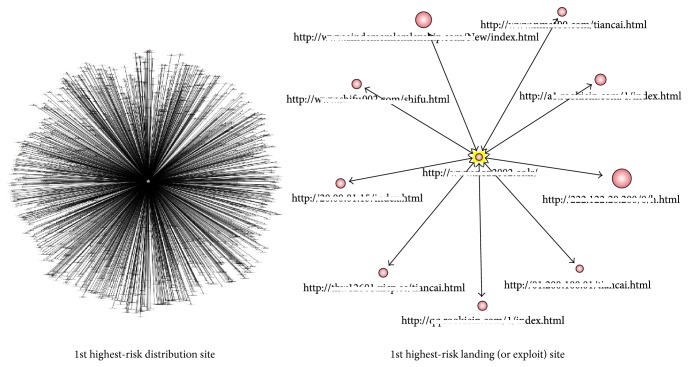
Visualization of malware site risk.

Figure 4 shows the MRI estimated through the 1st and 2nd order risk analysis after the detection of malicious URLs.

Table 1 lists the detected malicious code exploit/landing/distribution URLs (including both exploit/landing sites and distribution sites). The risk index is a relative value. If limited to the range 0-1, the minimum risk would be fixed at 0, but it is hard to set a clear standard for the maximum risk.

In this paper, we use a relative risk index that fixes the minimum risk to 0 and indicates the high-risk core malware sites through prioritization.

### 4.2. Sensitivity Analysis

The detection rate of actual zero-day attacks can be measured using a sensitivity analysis based on the results given in Table 1. Among the malware sites related to zero-day attacks occurring to biomedical information systems, we analyze distribution sites and exploit/landing sites.  Table 2 shows the detection rate measurements based on actual data produced in a specific time window.

The results in Table 2 focus on the top five high-risk sites. The multipath malware site group denotes the number of exploit/landing sites actually connected with a distribution site. The percentage represents the average detection rate in a specific time window, and this detection performance is better than in the pre-analysis stage.

The average early detection rate of distribution sites and exploit/landing sites is also higher in this section than in the preanalysis stage. That is, the proactive elimination of core malicious websites results in an average improvement in zero-day attack detection of more than 20%.

### 4.3. Visualization of Analysis Results

The risk index of each URL calculated in this paper can be analyzed by verifying whether the risk index agrees with the weak point of the corresponding server.

This section analyzes the actual weak point based on the calculated risk index and verifies whether this index agrees with the actual prioritization using an error analysis technique. Figure 4 visualizes the 1st highest-risk distribution site according to the MRI.

The detection and elimination of high-risk malicious code exploit/landing/distribution sites related to biomedical information systems can be achieved by visualizing the 1st highest-risk exploit/landing/distribution site, as shown in Figure 4. Thus, our proposed model focuses on estimating the risk presented by target malware sites in the specific field of biomedical information.

We verify the performance of the proposed model based on static analysis. However, for military, government, or similar organizations, we must dynamically filter out core malware sites based on high-performance hardware platforms. For this reason, our method is a good example of a suitable defensive measure for APT attacks.

## 5. Related Work

Methods for detecting and analyzing websites including malicious code can generally be divided into static and dynamic analysis.

### 5.1. Static Analysis

Static analysis mainly uses machine learning and pattern matching to detect and classify malicious URLs.

Ma et al. [14, 15] presented a classification model that detects spam and phishing URLs. This model uses a statistical method to classify URLs by considering the lexical and host-based properties of malicious URLs. Although this method detects both spam and phishing URLs, it cannot distinguish between the two.

Another approach is to analyze the JavaScript code in web pages to find the typical features of malicious code. This is done either statically [16] or dynamically by loading the affected pages in an emulated browser [17]. Systems such as Prophiler [18] consider both JavaScript and other features found in HTML and the URLs of malicious pages. Whittaker et al. [19] proposed a phishing website classifier to automatically update Google's phishing blacklist. They used several features obtained from domain information and page contents.

JSAND [20] used a machine learning approach to classify malicious JavaScript.

### 5.2. Dynamic Analysis

Dynamic analysis analyzes the server–client connection to detect and classify malicious URLs.

In other words, dynamic analysis relies on visiting websites with an instrumented browser (often referred to as a honeyclient) and monitoring the activities of the machine to find the typical signatures of successful exploitations (e.g., the creation of a new process) [21]. PhoneyC [22] uses a signature-based low-interaction honeypot to detect malicious websites.

Systems such as [23, 24] execute web content dynamically and capture drive-by downloads based on either signatures or anomaly detection, while Blade [25] leverages user behavior models for drive-by download detection. All of these systems exhibit good detection results. However, it is usually costly to follow the full redirection path and monitor each script execution in real time. Moreover, their accuracy is highly dependent on the malicious response of the webpage to vulnerable components.

Provos et al. [26] analyzed the maliciousness of a large collection of web pages using a machine learning algorithm as a prefilter for VM-based analysis. They adopted content-based features including the presence of obfuscated JavaScript and exploit site-pointing iframes.

The main differences between the models proposed in this paper and previous approaches are as follows.

- The model proposed in this paper applies a static method to analyze the connectivity between nodes and detects the core-hub node dynamically based on the risk index.
- The proposed model detects and blocks the core-hub node using link data from the high-risk malicious websites as observed for a specific period of time.
- The proposed model prevents the dissemination of malicious websites in the early stages by blocking the link between the core malicious code distribution site and the exploit/landing site.

## 6. Conclusion

In this paper, the 1st order risk of malware infection was analyzed using log information estimated by an MCC that considers the DCI, BCI, and ECI of the main nodes based on the priority of risk. This provides a quantitative value of the potential risk inherent in the corresponding site (node).

In addition, the risk index of exploit sites and distribution sites was calculated by considering their weights. The overlapped infection history and survival ratio were used to estimate the risk of distribution sites, whereas the overlapped infection history and exposure frequency were considered when estimating the risk of exploit sites. Finally, the MRI was estimated using the 1st order risk analysis and the risk index of the distribution sites and exploit sites.

In future work, we will develop a feature model that predicts the seriousness of website security problems by data-mining the logs produced from malicious code detection and vulnerability scanning tools.

As this feature model will be used to predict the risk of a specific website, it should contribute to establish an active malicious code distribution blocking system that realizes proactive responses beyond the limit of reactive responses that rely only on traditional malicious code detection tools.

## Figures and Tables

**Table 1:** MRI estimation result of exploit/landing/distribution sites.

Node type	URL	MRI	Reliability
Distribution site	http://222.∗∗∗.∗∗∗.∗∗∗/c/h.html	0.3965	91%
Distribution site	http://www.∗∗∗∗∗∗∗∗∗.com/New/index.html	0.3505	92%
Distribution site	http://a1∗∗∗∗∗∗∗∗∗.com/1/index.html	0.3058	90%
Exploit site	http://www.∗∗∗∗∗∗∗∗∗.or.kr/	0.3047	95%
Exploit site	http://www.∗∗∗∗∗∗∗∗∗.co.kr/	0.3026	94%
Exploit site	http://www.∗∗∗∗∗∗∗∗∗.kr/	0.3017	94%
Distribution site	http://a2.∗∗∗∗∗∗∗∗∗.com/2/index.html	0.3009	93%
Exploit site	http://www.∗∗∗∗∗∗∗∗∗.re.kr/	0.3003	92%
Exploit site	http://www.∗∗∗∗∗∗∗∗∗.or.kr/	0.2993	92%
Exploit site	http://www.∗∗∗∗∗∗∗∗∗.co.kr/	0.2991	90%
Exploit site	http://www.∗∗∗∗∗∗∗∗∗.co.kr/	0.2983	91%
Exploit site	http://www.∗∗∗∗∗∗∗∗∗.co.kr/	0.2982	90%
Exploit site	http://www.∗∗∗∗∗∗∗∗∗.org/	0.2970	94%
Exploit site	http://www.∗∗∗∗∗∗∗∗∗.or.kr/	0.2969	96%
Exploit site	http://www.∗∗∗∗∗∗∗∗∗.com/	0.2968	95%
Exploit site	http://∗∗∗∗∗∗∗∗∗.co.kr/	0.2967	95%
Exploit site	http://www.∗∗∗∗∗∗∗∗∗.co.kr/	0.2966	96%
Exploit site	http://www.∗∗∗∗∗∗∗∗∗.kr/	0.2966	94%
Exploit site	http://www.∗∗∗∗∗∗∗∗∗.kr/	0.2962	93%
Exploit site	http://www.∗∗∗∗∗∗∗∗∗.com/	0.2961	93%
Exploit site	http://www.∗∗∗∗∗∗∗∗∗.co.kr/	0.2960	94%

(“∗∗∗∗∗∗∗∗∗”: the URL information of malware site).

**Table 2:** Average detection rate of zero-day attacks for a given day.

Priority of risk	Malware site group with multipath	Distribution site with single path	Landing (or exploit) site with single path
1	23.3% (15)	21.5%	28.2%
2	22.6% (9)	31.6%	32.4%
3	14.7% (8)	22.8%	18.1%
4	18.4% (10)	19.7%	32.3%
5	21.2% (12)	24.2%	17.6%
Average early detection rate	20.04%	23.96%	25.72%
